# How Do You Play? A Comparison among Children Aged 4–10

**DOI:** 10.3389/fpsyg.2016.01833

**Published:** 2016-11-17

**Authors:** Elisa Delvecchio, Jian-Bin Li, Chiara Pazzagli, Adriana Lis, Claudia Mazzeschi

**Affiliations:** ^1^Department of Philosophy, Social Sciences and Education, University of PerugiaPerugia, Italy; ^2^Department of Developmental Psychology and Socialization, University of PadovaPadova, Italy

**Keywords:** affect in play scale-preschool version, affect in play scale-preschool extended version, construct validity, divergent thinking, Italian children

## Abstract

Pretend play has a central role for children's development and psychological well-being. However, there is a paucity of standardized and valid measures specifically devoted to assess the core domains involved in play activities in preschool and primary school children. The Affect in Play Scale-Preschool (4–5 years) and the Affect in Play Scale-Preschool Extended Version (6–10 years) are semi-structured parallel tools designed to explore child's cognitive and affective processes using a standardized play task. The current study administered this 5-min play task to 538 Italian children aged 4–10. The purposes were to compare play abilities in boys vs. girls and in preschool vs. primary school children, to correlate pretend play with divergent thinking and to evaluate the structural validity of the measure along the considered age span. No differences, excepting for Organization, were found between boys and girls, whereas school age children reported higher play abilities then the younger ones. External validity was assessed using correlational analysis with the divergent thinking task (the Alternate Uses Test) for preschoolers and primary school-aged children, in line with findings from Manova. Construct validity, assessed through the Confirmatory Factor Analysis, showed good fits for the two-factor model with cognitive and affective factor for both the Affect in Play Scale-Preschool and its Extended Version. A multi-group factor analysis suggested a partial invariance of the two-factor model across preschool (4–5 years old) and primary school-aged (6–10 years old) children. Results supported the use of the Affect in Play Scale-Preschool and its Extended Version as adequate measures to assess the interplay of cognitive and affective skills in preschool and school age children. The discussion highlights clinical and research implications linked to the possibility to have a unique play task able to assess child's affective and cognitive abilities throughout a quite wide life span (from 4 to 10 years old).

## Introduction

Pretend play represents symbolic behavior in which “one thing is playfully treated as if it were something else” (Fein, 1987). Pretend play is characterized by children's exploration and interpretation of the world in terms of symbols and images, fantasy, make-believes, expression of emotions, and their capacity to understand different situations in an imaginary context (Russ, 2004). Pretend play allows the child to comprehend and discover his world, talk about emotions, and integrate internal states and external actions (Stagnitti et al., 2007). In pretend play, children have the opportunity to act out social situations that facilitate their understanding of the world in which they live (Bundy-Myrow, 2005; Ferland, 2005; Moore and Russ, 2006). As such, pretend play could be thought as “practice for real life.” Pretend play represents the integration of cognitive and affective processes. Cognitive processes comprehend, among others, divergent thinking, symbolism, and a fluent organization of stories. Affective processes include expression of emotions and affect themes in the play story.

Pretend play peaks and takes on special significance in preschool years (Singer and Singer, 1990; Bergen, 2002; Pellegrini, 2010). During these years, pretend play has been associated with language, as well as, emotional and social abilities development (Westby, 2000; Uren and Stagnitti, 2009). However, pretend play assumes a very important role also in elementary school-age years. Pretend play in preschool and school years children was related to divergent thinking (Russ and Schafer, 2006; Delvecchio et al., 2016b), coping ability (Christiano and Russ, 1996), problem solving and adjustment (Russ, 2004, 2006), perspective taking and emotional understanding (Seja and Russ, 1999; Fehr and Russ, 2014), empathy (Delvecchio et al., 2016a), and self-rated emotional experience (Russ and Grossman-McKee, 1990; Hoffmann and Russ, 2012; Russ, 2014) and growing capacity of self-regulation (Berk et al., 2006). Thus, having valid and reliable measures to assess pretend play abilities in children it's necessary (Bergen, 2002; Pellegrini, 2010).

Previous studies highlighted the paucity of standardized tools for pretend play and stressed that most of the existing ones are not able to assess child's development in a broad manner, paying attention to both cognitive and affective aspects (Delvecchio et al., 2016b). To date the Affect in Play Scale (APS; Russ, 1993, 2004) and its Preschool version (APS-P; Kaugars and Russ, 2009) are two of the few valid and empirically-based pretend play measures that assess both cognitive and affective processes involved in play. An increasing number of research studies have supported the adequacy of their psychometric characteristics in both the United States and in Italy (Russ, 2004; Mazzeschi et al., 2008; Delvecchio et al., 2016b). However, the different stimuli of APS (puppets) and APS-P (toys) and the different age-group involved (i.e., 4–5 years old for APS-P and 6–10 for APS) emphasize the difficulties in having a valid instrument able to compare preschool and school-aged children's pretend play abilities. Recently, to overcome this issue, Delvecchio et al. (2016a) adapted the APS-P (APS-P Extended Version) for primary school children, motivating this extension as the following: (1) school-aged children often play in a more joyful way with toys than puppets (Mazzeschi et al., 2008); (2) having a tool able to compare children throughout a wider age-span would be a useful improvement for both clinical (e.g., for children with developmental delay) and research settings. The first Italian research on psychometric properties of APS-P and APS-P Extended Version evidences promising results (Delvecchio et al., 2016a,b). More specifically, confirmatory factor analysis (CFA) showed the adequacy of the APS-P original model (Russ, 2004) which includes two correlated factors: one cognitive (organization, elaboration, imagination, and comfort) and one affective factor (frequency and variety of affects) for both the preschool and its extended versions. External validity was proved correlating the play task with measures of divergent thinking, children's temperament, prosocial behavior, school coping, and empathy (Delvecchio et al., 2016a,b). The APS-P and the APS-P Extended Version share the same stimuli and scoring system and they attempt to fill the gap of “discontinuity” between the age-span.

The aim of this paper is to investigate the use of this “common” tool in a sample of preschool and primary school children. This paper investigated reliability and validity of the APS-P and APS-P Extended Version with Italian children aged 4–10. As preliminary result, interrater reliability was assessed and expected to be excellent, as previous studies found (Delvecchio et al., 2016a,b). Descriptive statistics for the overall sample and separately for boys and girls as well as for preschool and school children were calculated. According to previous studies on playing no significant gender differences were expected (Stagnitti et al., 2007; Kaugars and Russ, 2009). However, significant differences were expected between preschool and school age children (Mazzeschi et al., 2008; Chessa et al., 2011). It was expected that elementary school children showed higher abilities in organizing and elaborating the stories; they were expected to use more fantasy and to express higher comfort during the play task. At the same time, they were supposed to use a higher amount of affect expressions and a wider variety of categories of affective themes suggesting that school age may possess a greater use and comprehension of emotions (Denham et al., 1994). Moreover, external validation of the APS-P and its extended version variables was carried out by investigating its relationships with the divergent thinking. Significant correlations, with medium to low effect sizes, were expected. Then, a confirmatory factor analysis was run separately for preschool and primary school children to assess the adequacy of the two-factor model with a cognitive and affective correlated factors, as previously found in Italian preschool (Delvecchio et al., 2016b) and primary school children (Delvecchio et al., 2016a). Finally, a multi-group confirmatory factor analysis (MCFA) was run on APS–P and APS-P Extended Version, looking for the measurement invariance of a two factor model between preschool and primary school children (Russ, 2004; Chessa et al., 2011; Delvecchio et al., 2016a,b).

## Materials and methods

### Participants

A sample of 538 Italian children (261 boys, 277 girls) aged 4–10 years (*M* = 6.61, *SD* = 2.20) were enrolled in this study. Ten kindergartens and 8 primary schools from urban areas of Central and Northern Italy were contacted. Specifically, 239 children (44.4%) were preschoolers whereas 299 (55.8%) came from elementary schools. All children were Caucasian and were in mainstream classrooms. Family socioeconomic status was measured using the SES scores by Hollingshead (1975) and showed a majority of middle-class (i.e., SES level 3) families, 16% came from a high socioeconomic context (i.e., SES levels 4 and 5) and 4% were from economically disadvantaged families (i.e., SES levels 1 and 2). Children' distribution in gender, age, and SES was not significant different from the overall sample. According to their teachers, all children were developing typically. Before starting the task, preschoolers' cognitive and verbal skills were assessed using two subtests (vocabulary and bloc design) of the WPPSI-III (Wechsler, 2008). In order to participate in the study, a score higher than 8 was mandatory. At the same time, for primary school children, cognitive, and verbal abilities were confirmed by teachers' reports on the basis of Italian language and maths tests.

### Procedure

This study was conducted in compliance with the ethical standards for research outlined in the Ethical Principles of Psychologists and Code of Conduct (American Psychological Association, 2010) and approved by the Ethical Committee of the Department of Psychology of the University of Padova (Italy). Participation in the study was solicited via leaflets. School approval and parents written signed informed consent to participate in the study were obtained before data collection. Children were asked to provide their own oral consent. No incentives were awarded and voluntary participation was emphasized. Administration was proposed during scheduled classes, according to the standard administration procedures. Confidentiality was assured by replacing children's personal information with a numeric code.

### Measures

#### Affect in play scale-preschool and affect in play scale-preschool extended version

The Affect in Play Scale-Preschool Version (Russ, 2004; Kaugars and Russ, 2009), and its extended version (Delvecchio et al., 2016a), are 5 min video-recorded play task assessing child's affective and cognitive dimensions, using a standardized and empirically validated administration procedure and scoring attribution (Russ, 2004, p. 19). Children to accomplish the task should play with a set of stuffed and plastic toys (Russ, 2004), specifically selected to elicit a range of different emotional expression. Although, the stimuli and scoring system did not change from APS-P to APS-P Extended Version, the instructions for the APS-P Extended Version were slightly modified avoiding direct speech and using the indirect one (for detail see Delvecchio et al., 2016a). APS-P and APS-P Extended Version (4–10) scoring system comprehends six primary scores: Organization, Elaboration, Imagination and Comfort, Variety and Frequency of Affective Themes. The first four scores refer to the cognitive dimension, while the last two belong to the affective domain and the emotional expression of play. Cognitive scores are coded on a 5-point Likert scale, with higher values suggesting higher play abilities. Organization assesses the quality of the plot, the complexity of the story, and the coherence of the play narrative; Elaboration assesses the amount of variety and complexity of embellishment in the plot, facial expressions, sound effects, the toys used, and character development; Imagination refers to the novelty and uniqueness of the play and the ability to pretend and use fantasy, the ability to transform the blocks and pretend with them and Comfort measures the child's ability to engage in the play task, the involvement of the child in the play and enjoyment of the play. Referring to the affective domain, Frequency of Affect Expression, counts affects expressed by the child in the play narrative, whereas the Variety of Affect score is the number of different affect categories expressed by the child during the play. Beside the six main scores, four minor ones are available: Positive affect score including themes of nurturance/affection, happiness/pleasure, competition, oral, sexual; Negative affect one referring to aggression, anxiety/fear, sadness/hurt, frustration/disappointment/dislike, oral aggression, anal. Furthermore, starting from the Variety of Affect score is possible to calculate Positive affect variety as well as the Negative affect variety scores. Interrater reliability reported excellent values as well as the internal consistency of the scales (Kaugars and Russ, 2009; Chessa et al., 2011; Fehr and Russ, 2014; Delvecchio et al., 2016a,b).

#### Alternate uses task assesses

Divergent Thinking based on Wallach and Kogan's (1965) adaptation of Guilford's Alternate Uses Task. The task includes a list of six common objects (a newspaper, a box, a button, a key, an automobile tire, a shoe, and a knife) and requires telling as many alternative uses for those objects. Alternate uses task scoring system comprehends two scores: (1) Fluency referring to the amount of acceptable possible uses listed and (2) Flexibility referring to the amount of different categories of acceptable possible uses listed by the child. Psychometric characteristics of the Alternate Uses Task have been reported by Kogan (1983) and Runco (1991).

### Data analysis

Interrater reliability among the examiners was assessed using the interclass correlation coefficient with a 95% confidence interval. Descriptive statistics for APS-P and APS-P Extended Version variables were calculated. Analysis of variance (ANOVA and MANOVA) was used to assess the possible effects of preschool vs. primary school children and gender. Partial eta squared was employed to evaluate effect size (Cohen, 1988; Richardson, 2011). Pearson product-moment correlations were calculated to examine the strength of the correlation between the APS–P, APS-P Extended Version, and the Alternate use test. Finally, Confirmatory Factor Analysis (CFA) was carried out on preschool and school-aged children separately. Multi-group confirmatory factor analyses (MCFA) were run with increasing parameter constraints to assess the measurement invariance of APS-P across age. Configural invariance (equivalence of the factorial structure) of the hypothetical model (baseline model) was performed. After that, metric invariance (equivalence of the factor loadings), and scalar invariance (equivalence of the factor loadings and the intercepts of items) were assessed. Measurement invariance was established when (a) BIC showed lower values and change in CFI (ΔCFI) was negligible (< 0.01) between increasingly constrained models (b) the other fit indexes of the model indicated a good fit (Vandenberg and Lance, 2000; Cheung and Rensvold, 2002; Chen, 2007; Thienot et al., 2014).

R (R Development Core Team, 2012) Package lavaan (Rosseel, 2012) and the PASW Statistics 18, (SPSS Inc., 2009) were selected to run the analyses.

## Results

Inter-rater reliability was calculated on 30 videos (random selection) for the APS-P and 30 for the APS-P Extended Version. Four judges scored independently the 60 protocols according to the manual (Russ, 2004; Kaugars and Russ, 2009). ICCs ranged from a minimum of 0.85 to a maximum of 0.91. The coders then independently scored about 120 protocols each.

Means and standard deviations for APS-P and APS-P Extended Version play scores are presented in Table 1. ANOVAS and MANOVAS were performed to examine gender and age-related difference for cognitive and affective scores (Table 2).

**Table 1:** **Descriptive statistics for APS-P and APS-P Extended Version for the total sample, boys, girls, preschoolers and school-aged children**.

	**Total (*N* = 538)**		**Gender**				**Age**			
	***M***	***SD***	**Boys (*n* = 261)**		**Girls (*n* = 277)**		**Preschool (*n* = 239)**		**School (*n* = 299)**	
			***M***	***S*D**	***M***	***SD***	***M***	***SD***	***M***	***SD***
Organization	2.40	0.99	2.31	1.01	2.49	0.97	1.89	0.85	2.82	0.91
Elaboration	2.36	1.10	2.25	1.12	2.45	1.07	2.08	1.09	2.57	1.06
Imagination	2.42	0.92	2.41	0.95	2.44	0.90	2.05	0.88	2.72	0.84
Comfort	3.28	0.10	3.20	0.96	3.35	0.85	3.14	0.96	3.38	0.85
Frequency of affect expression	29.90	19.14	28.80	18.69	30.94	19.53	24.70	20.07	34.06	17.31
Positive affect	18.10	13.34	15.90	12.79	20.15	13.54	12.80	11.87	22.31	12.96
Negative affect	7.96	7.90	8.69	8.02	7.27	7.76	6.59	7.92	9.05	7.74
Variety of affect	5.00	2.22	5.05	2.28	4.96	2.18	4.15	2.30	5.70	1.91
Positive variety of affect	2.50	1.06	2.41	1.14	2.57	0.980	2.13	1.17	2.13	1.17
Negative variety of affect	2.50	1.56	2.70	1.54	2.40	1.56	2.02	1.51	2.88	1.47

**Table 2:** **Analysis of variance for gender and age**.

		***F*_(1, 534)_**	***p***	**η^2^**
Organization	Gender	5.33	0.021	0.009
	Age	148.31	< 0.001	0.217
Elaboration	Gender	4.19	0.041	0.008
	Age	27.30	< 0.001	0.049
Imagination	Gender	0.18	0.673	0.001
	Age	81.47	< 0.001	0.132
Comfort	Gender	3.54	0.060	0.007
	Age	9.59	0.002	0.018
Frequency of affect expression	Gender	1.38	0.241	0.003
	Age	33.20	< 0.001	0.059
Positive affect	Gender	13.92	< 0.001	0.026
	Age	77.44	< 0.001	0.127
Negative affect	Gender	4.25	0.040	0.008
	Age	13.51	< 0.000	0.025
Total variety of affect	Gender	0.25	0.614	0.001
	Age	73.32	< 0.001	0.121
Positive variety of affect	Gender	2.56	0.134	0.004
	Age	63.12	< 0.001	0.106
Negative variety of affect	Gender	2.95	0.087	0.005
	Age	44.81	< 0.001	0.078

### Gender differences

A significant effect of gender emerged for Organization with girls (*M* = 2.49, *SD* = 0.97) scoring higher than boys (*M* = 2.31, *SD* = 1.01). Although, gender turned out to be significant also for Elaboration, with girls (*M* = 2.45, *SD* = 1.07) reporting higher values than boys (*M* = 2.25, *SD* = 1.12), the partial eta-squared estimates were low. Thus, only trivial effects, mainly due to the large sample size, can be hypothesized. No significant differences were found for Imagination and Comfort. Referring to affect variables, no significant differences were found for the Total Frequency of Affect Expression neither for the Total Variety of Affect. However, looking at subscales, Frequency of Positive Affect evidenced a larger amount of those themes in girls (*M* = 20.15, *SD* = 13.54) than in boys (*M* = 15.90, *SD* = 12.70). Although, significant, the Frequency of Negative Affect reported negligible value of the partial eta-squared estimate. No significant differences were found for Variety of Positive, as well as Negative Affect.

### Age-related differences

Significant differences were found for preschool vs. primary school children for Organization, Elaboration, Imagination, and Comfort (Table 2), with older children scoring higher than preschoolers (Table 1). No significant interactions emerged. Looking at affective scores, significant differences were found for all the variables with preschool children reporting lower number and variety of affective expressions. No significant influences were found for interaction.

Table 3 shows Pearson's correlations between play task (cognitive and affective factors) and measures of divergent thinking. Significant correlations between cognitive and affective scores on the APS-P and APS-P Extended Versions with divergent thinking were found, with the only exception of the Frequency of Negative Affect and the two scores of divergent thinking. In general, children who demonstrated greater abilities in cognitive and affective play abilities showed higher score in divergent thinking (both in Fluency and Flexibility).

**Table 3:** **Correlations between APS-P, APS-P Extended Version and Divergent Thinking for total sample, preschool, and primary school children**.

		**Fluency**	**Flexibility**
Total sample (*N* = 538)	Organization	0.23^**^	0.30^**^
	Elaboration	0.13^**^	0.19^**^
	Imagination	0.13^**^	0.29^**^
	Comfort	0.15^**^	0.17^**^
	Frequency of affect expression	0.15^**^	0.10^*^
	Positive affect	0.16^**^	0.11^*^
	Negative affect	0.07	0.07
	Total variety of affect	0.16^**^	0.16^**^
	Positive variety of affect	0.12^**^	0.16^**^
	Negative variety of affect	0.14^**^	0.13^**^
Preschool children (*n* = 239)	Organization	0.16^**^	0.25^**^
	Elaboration	0.17^**^	0.23^**^
	Imagination	0.07	0.19^**^
	Comfort	0.15^*^	0.22^**^
	Frequency of affect expression	0.27^**^	0.11
	Positive affect	0.25^**^	0.06
	Negative affect	0.17^**^	0.12
	Total variety of affect	0.16^**^	0.17^*^
	Positive variety of affect	0.10	0.16^*^
	Negative variety of affect	0.10	0.16^*^
Primary school children (*n* = 299)	Organization	0.23^**^	0.28^**^
	Elaboration	0.07	0.11^*^
	Imagination	0.10	0.16^*^
	Comfort	0.13^*^	0.10
	Frequency of affect expression	0.01	0.03
	Positive affect	0.05	0.06
	Negative affect	−0.06	−0.05
	Total variety of affect	0.11	0.09
	Positive variety of affect	0.09	0.09
	Negative variety of affect	0.09	0.06

* p < 0.05;

** p < 0.01.

However, in line with findings from the MANOVA, correlations were run for preschoolers and school-aged children separately (Table 3). Results showed significant correlations, although with low effect sizes, between Fluency and most of the APS-P variables, excepting for Imagination and Variety of Negative affect for preschool children, whereas looking at school-aged children, the only two significant correlations were found for Organization and Comfort. Looking at Flexibility, positive significant correlations were found only for the cognitive scores of APS-P. A similar pattern was seen in older children (APS-P Extended Version), who showed significant positive correlations between Flexibility and Organization, Elaboration and Imagination. No significant correlation emerged between Flexibility and Comfort, as well as the affective scores.

Fit indices of CFAs carried out for preschoolers and primary school children separately reflected a good fit (Table 4). Fit indices of the measurement invariance procedure are reported in Table 4. The configural model (M1) showed adequate fit, prompting for the equivalence of the factorial structure of the construct across groups. Shifting to metric invariance (M2), the value of the ΔCFI was negligible and the BIC was lower (Table 4). However, a different pattern was found when testing for scalar invariance (M3). In this case fit was poor, value of the ΔCFI was greater than the recommended cut off and BIC was higher. Therefore, these findings provided weak evidence of measurement invariance for the two factor theoretical model across preschool and school-aged children. In such cases, as suggested by van de Schoot et al. (2012), there is the need to test for partial invariance by freeing one item at the time. According to Dimitrov's (2010) guidelines, < 20% free parameters are acceptable, which represent < 2 items in the case of the APS-P. Inspection of the modification indices suggested that freeing the intercepts of item 1 (Organization) across groups would provide a better values. Therefore, the partial scalar invariance model (M3a) was run. It showed an adequate fit. Results displayed evidence of partial invariance between the groups showing acceptable values of ΔCFI and a lower BIC value (Table 4) between M3a and M2.

**Table 4:** **Test of measurement invariance of the APS-P and APS-P Extended Version across age (*N* = 538)**.

	**χ^2^**	***df***	***p***	**RMSEA**	**TLI**	**CFI**	**ΔCFI**	**BIC**
Baseline model: preschoolers (*n* = 239)	20.68	8	0.008	0.081	0.985	0.987		
Baseline model: school-aged (*n* = 299)	23.41	8	0.003	0.080	0.956	0.977		
M1: configural invariance	44.09	16	< 0.001	0.081	0.967	0.983		11378.21
M2: metric invariance	64.57	20	< 0.001	0.091	0.958	0.976	0.007	11373.54
M3: scalar invariance	162.32	24	< 0.001	0.146	0.892	0.914	0.062	11446.12
M3a: partial scalar invariance	101.43	23	< 0.001	0.094	0.936	0.967	0.009	11371.53

RMSEA, Root Mean Square Error of Approximation; TLI, Tucker-Lewis Index,; CFI, Comparative Fit Index; BIC Bayesian Information Criterion.

## Discussion

This study investigated reliability, gender and age-related differences and validity of the APS-P and APS-P Extended Version administered to a community sample of preschool and primary school children. APS-P Extended Version is an extension of APS-P, a measure of preschool children's play, to school age children, aimed to facilitate the comparison of children cognitive and affective interplay components in pretend play.

Although, some significant gender differences were found for some of the cognitive and affective variables, eta squared magnitudes were small, prompting for trivial results. These findings replicated Kaugars and Russ' (2009) research in which they did not find any gender differences. However, significant differences were found between preschool and school-aged children for both the cognitive and affective variables. Elementary school children appeared significantly more able to organize their play, to design more sophisticated plot, to produce more complex story, and to have higher coherence in the play narrative (Organization). They also appeared significantly more able to use pretend and fantasy to transform a thing as another, to add elements outside of daily experience, to show more novelty of ideas (Imagination). Moreover, preschool and school children differed on the amount of variety and complexity of embellishment in the story themes, facial expressions, toys chosen for play, and character description (Elaboration) with older children scoring higher then youngers, as well as it was found for their comfort in play. Furthermore, school-aged children expressed a significant larger amount of affective expressions (both positive and negative) and used them in a wider variety, suggesting that older children, in line with their development, may have a better knowledge, understanding and expression of emotions then preschoolers (Denham et al., 1994).

Significant correlations between APS-P and its extended version cognitive and affective scores and divergent thinking were found, showing children with greater play abilities scoring higher in both Fluency and Flexibility. Previous research has already documented that cognitive as well as affective components of pretend play are linked to higher abilities in divergent thinking (Kelly-Vance et al., 2002; Butcher and Niec, 2005; Russ and Schafer, 2006; Marcelo and Yates, 2014). However, results of correlations carried out for preschool and school-aged children evidenced age-related differences. The preschoolers sample showed a larger amount of significant correlations between play abilities (both cognitive and affective domains) and the capacity to generate acceptable uses for one object (Fluency). Delvecchio et al. (2016a), found similar results in their study on children aged 6 to 10 years. No such age-discrepancies were found between APS-P/APS-P Extended Version and Flexibility. Moreover, some of the correlations showed a low effect size. In other words, as expected, external criteria accounted only for a portion of the variance of the cognitive and affective scores but not for the entire relationship.

CFAs results supported the best data fit for the theoretical two-correlated-factor model involving two factors related to cognitive and affective dimensions for both samples. As postulated theoretically by Kaugars and Russ (2009) and empirically by Delvecchio et al. (2016a,b), the cognitive factor was significantly loaded by the four cognitive scores, whereas the affective one by the emotion-related variables (i.e., Total Frequency of Affect Expression and Total Variety of Affect). MCFA showed some evidence of measurement consistency across preschool and school-aged children; metric invariance was partial and obtained after freeing organization variable.

Although, the present study was conducted on a quite large community sample of Italian preschool and primary school children, limitations should be noted. Because this is the first study comparing results on the APS-P and APS-P Extended Version, findings need to be replicated in future research with national as well international groups. Generalizability of the findings is limited to the Italian sample. Cross-cultural studies should focus on similarities and/or differences in play taking into account children's cultural background. Previous studies have already pointed out the importance of specific cultural parenting and educational practices in increasing cognitive or affective expression during pretending (Chessa et al., 2012). However, cross-cultural research on APS-P, comparing samples from Italy and the US, suggests a quite high correspondence in play abilities across countries (Chessa et al., 2012). One more limitation regards the socioeconomic status that in this sample was medium, although, as suggested by Russ (2004), there is no reason to expect that findings would be different for children of a different socioeconomic status.

Second, the low to medium effect sizes magnitudes of the correlations, although in line with previous findings, warrant further research. Third, external validity was checked just with a creativity measure, so further more studies are needed. However, previous study on APS-P Extended Version in school-aged children showed wider evidences on external validity of the scale (Delvecchio et al., 2016a). Fourth, this study enrolled a sample of non-clinical children. Further studies should be carried out to compare normative and clinical samples of children with different diagnoses. It should be useful to detect possible differences in pretending for different disorders. Fifth, parental and teachers ratings of children's behaviors or affect regulation might also prove useful in future studies. The analysis of different informants' perspectives will enlarge the focus of investigation and provide a more complete picture of the child (Achenbach, 2006).

During the decades play has been considered as a milestone for children growth. Several studies pointed out how play could promote cognitive development, increase creative processes, enhance problem solving, divergent thinking, self-regulation, coping, positive relationships, and can manage social settings to understand the world (Russ, 2004; Bundy-Myrow, 2005; Colwell and Lindsey, 2005; Ferland, 2005; Moore and Russ, 2006; Uren and Stagnitti, 2009). International literature has pointed out how play is an important tool for children's assessment because it emphasizes developmental cognitive, emotional and social acquisitions (Stagnitti, 2004; Russ, 2012). Play is considered an important tool for allowing adaptation and the processing of reality (Capurso and Pazzagli, 2016). However, empirical studies are not yet as well-established as in the clinical and observational tradition (O'Connor and Stagnitti, 2011). Assessment of play along preschool and school age can be useful in longitudinal studies, but also in pre-post intervention with elementary school children with psychological difficulties or disadvantaged backgrounds, where their level of scores could not be foresee. Although, the role of pretend play in longitudinal research and the assessment of pre-post therapeutic interventions has been recognized, emphasized, and empirically studied by several authors (Russ, 2004; Moore and Russ, 2006; Hoffmann and Russ, 2016) none of these studies adopted the APS-P or APS-P Extended Version. Thus, further research is needed.

In conclusion, the results of this study suggest that APS-P and APS-P Extended Version lead to scores that are reliable, are related to external criteria similarly and have a similar underlying factor structure as the original APS-P scale and are suitable for children from 4 to 10 years old.

## Author contributions

ED contributed to conception and design, on acquisition of data and on drafting of manuscript; JL contributed on analysis and interpretation of data; CP contributed on drafting of manuscript; AL contributed to conception and design and revising it critically; CM contributed to conception and design and gave final approval of the version.

### Conflict of interest statement

The authors declare that the research was conducted in the absence of any commercial or financial relationships that could be construed as a potential conflict of interest. The reviewer DV and handling Editor declared their shared affiliation, and the handling Editor states that the process nevertheless met the standards of a fair and objective review.
